# Bridging knowledge gaps: identifying key educational priorities for healthcare professionals in the United Arab Emirates

**DOI:** 10.1186/s12909-026-09121-x

**Published:** 2026-04-23

**Authors:** Hatem Alameri, Majd Al Dameh, Ahmed Husseiny

**Affiliations:** 1 College of Medicine and Health Sciences, Khalifa University, Abu Dhabi, United Arab Emirates; 2 Department of Health, Abu Dhabi, United Arab Emirates

**Keywords:** Continuous medical education, Continuous professional development, United Arab Emirates, Needs assessment

## Abstract

**Introduction:**

Healthcare professionals’ continuing medical education (CME) and continuing professional development (CPD) are essential for maintaining quality care. This study aimed to identify the perceived CME/CPD needs of healthcare professionals in the United Arab Emirates and explore barriers to professional development.

**Methods:**

A cross-sectional survey was distributed to all healthcare professionals who participated in CME/CPD activities in Abu Dhabi during 2022. The survey assessed learning experiences, barriers to participation, and perceived priority needs across clinical and professional domains. Descriptive statistics and chi-square tests were employed for analysis.

**Results:**

From 2,620 respondents (75.9% nurses, 12.6% allied health, 11.5% physicians), management of communicable diseases emerged as the highest CME priority (39.1%), followed by pain management (27.7%) and procedural skills (25.5%). For CPD, the top needs included knowledge of the healthcare system (38.2%), communication skills (34.3%), and evidence-based practice (33.5%). The primary barrier to participation was difficulty taking time off (38.4%), with 92.1% preferring live training over recorded formats. Physicians uniquely prioritize medical research (33.0%) and academic medicine (21.0%) compared with other professionals.

**Conclusion:**

This first comprehensive needs assessment in the region reveals both shared and professional-specific learning priorities within Abu Dhabi’s diverse healthcare workforce. These findings provide a foundation for developing targeted educational initiatives that address identified needs while overcoming common barriers to participation.

**Supplementary Information:**

The online version contains supplementary material available at 10.1186/s12909-026-09121-x.

## Introduction

Continuing Medical Education (CME) refers to learning activities that focus on updating clinical knowledge and technical skills for healthcare professionals [1]. CME is considered a vital subset of Continuing Professional Development (CPD), which includes both clinical and non-clinical competencies such as leadership and communication. While CME targets medical advances and practice updates, CPD encompasses lifelong professional growth. Both are essential for safe, effective clinical practice. CME/CPD credits are typically earned through various activities, such as attending medical conferences, participating in professional meetings, engaging in small group learning activities, and utilizing online e-learning resources [2]. These credits are essential for maintaining and enhancing professional competence in the medical field [3]. Although participation in CME/CPD has become a requirement for maintaining licensure and revalidating certification by numerous regulatory bodies, this does not appear to be the main incentive for professionals who participate in continuing education programs [4]. Daily clinical experiences are intricately linked to healthcare professionals’ motivation to learn. The challenges and issues encountered in practice are primary drivers for their ongoing education and skill development. This intrinsic motivation to learn is not only crucial for individual professional growth but also plays a vital role in maintaining societal trust in healthcare provision [5]. Real-world clinical scenarios drive the continuous acquisition of knowledge and skills, forming the foundation of lifelong learning for healthcare professionals Educational needs assessment is a gap-analysis that compares current practice performance to accepted standards [6]. It is defined as the interests or perceived requirements of a targeted population, and it is measured through the evaluation of quantitative or qualitative practice matrix, performance data, service utilization, focus-group discussion, survey, or feedback [7]. Multiple studies [7, 8] show that assessing educational requirements is crucial for strategic decisions that enhance professional competencies and improve patient outcomes. Assessing learner needs constitutes an essential stage in developing effective education and training programs, and its purpose is to determine the disparity between what is known and what should be known. Without needs assessment and analysis, the educational program may result in poor engagement and low participation. Most importantly, it may have little or no influence on learners’ practice, as it may fail to identify or address important issues from the participants’ perspectives [9].

The UAE’s healthcare sector, marked by workforce diversity and evolving needs, is undergoing significant transformation. All healthcare professionals in the UAE are required to engage in CME or CPD activities and earn credits to renew their licenses and maintain clinical privileges. A recent study in Abu Dhabi, UAE, revealed a remarkable 171% increase in CME activities and a 21% rise in CME credited hours compared to previous years, reflecting significant engagement by healthcare professionals in education and efforts to redesign learning environments to meet organizational competency needs [6].

CME activities are provided by various organizations based on specific operational requirements. However, despite the diverse academic and clinical backgrounds of healthcare professionals in the UAE, no population-level research has assessed their self-perceived learning needs. This study is the first of its kind locally and regionally, aiming to identify the top CME and CPD needs as perceived by different categories of healthcare professionals in the UAE. Additionally, it explores the obstacles healthcare professionals face in pursuing learning and professional growth within their work environments.

## Methods

The study included healthcare professionals who participated in CME or CPD activities between January 1 and December 31, 2022. The protocol was submitted to the Medical Research and Development Division of the Department of Health Abu Dhabi (DOH), which granted the study exemption from full ethical review (Ref# DOH/MRDD/2023/1142). Because the study did not involve the collection of sensitive personal data and posed no ethical concerns, the requirement for obtaining informed consent was waived. Participants were selected from certified providers registered in the DOH CME providers’ database (Accela Licensing Data Management System). CME providers were asked to distribute the survey to licensed physicians, nurses, and allied health professionals in their databases who had submitted CME applications or maintained accredited activities during the study period. Individuals not listed in the database, inactive in the past twelve months, or who did not provide consent, were excluded. Data collection encompassed provider contact information, CME application records, activity accreditation records, and CME coordinator contacts. A modified version of a previously published questionnaire was developed following a comprehensive review of the literature, including validated national and international surveys. The investigators identified and incorporated CME and CPD domains most relevant to local practice and compatible with the educational needs of physicians, nurses, and allied health professionals. Content validity was established through critical appraisal and consensus by all three study authors [10]. Survey participation was voluntary, and respondent anonymity was maintained throughout the data collection process. No personally identifiable information was collected. Participants received clear assurances that data would be used exclusively for research purposes, with strict confidentiality measures implemented to protect all collected information. The survey evaluated learning experiences from the past year and identified priority CME and professional development needs. The survey assessed: (1) continuing education opportunities utilized; (2) availability factors; (3) perceived CME needs across 33 clinical areas; and (4) perceived CPD needs across 34 professional areas using Likert Scale. The survey was administered using a web-based tool (Microsoft Office Forms, Microsoft 365 for Enterprise 2022).

Between September 1 and November 1, 2023, all CME coordinators from registered providers with accredited CME activities in 2022 were contacted by the DOH with an invitation to participate. The sample was selected based on 2023 statistics of 63,700 licensed healthcare professionals in Abu Dhabi. Using standard sample size formulae for large populations (95% confidence level, 5% margin of error), the minimum target sample size was calculated to be 382 respondents. Survey invitations were distributed to maximize representation, with a DOH associate ensuring follow-up and regular email reminders. Data analysis utilized Microsoft Excel, SPSS, and Julius. Inferential analyses were conducted using chi‑square tests of independence to assess associations between professional group and key demographic characteristics, with a two‑sided *p*‑value < 0.05 defining statistical significance. The top five CME and CPD topics were ranked for each professional group and for the overall sample using percentages rather than absolute counts, thereby accounting for differences in group representation.

## Results

A total of 2,620 healthcare professionals participated in the survey, comprising 1,989 nurses (75.9%), 331 allied health professionals (AHP) (12.6%), and 300 physicians (11.5%). The demographic information and practice profiles of the respondents are summarized in Table 1. Female participants accounted for 68.8% (*n* = 1,803) of the overall sample and were significantly more prevalent among nurses and allied health professionals than among physicians, for whom women represented 41%. Regarding practice characteristics, 45.9% (*n* = 817) of respondents had more than ten years of practice experience, and 53.4% had worked within the Abu Dhabi health system for less than five years. Practice settings differed significantly between groups, with 76.4% (*n* = 2,002) of participants employed in hospital environments. Ambulatory care was represented by 10.3% of respondents working in outpatient clinic settings. Longitudinal care services, encompassing long-term care, home-based healthcare, and rehabilitation facilities, constituted 8.3% of the practice distribution. The remaining 5.0% of healthcare professionals practiced in multispecialty medical and surgical centers. Concerning CME modalities, 48.5% of participants primarily engaged in live virtual attendance, while 43.6% predominantly attended live, in-person sessions. Enduring materials were the primary CME format for 7.9% of respondents. There were statistically significant differences in the types of CME attended across the three professional groups.

**Table 1:** Demographic characteristics, practice profiles, and barriers to CME/CPD participation among surveyed healthcare professionals (*N* = 2,620)

Variables	Total *n* = 2620 Actual %	Nurses *n* = 1989 (%)	Allied Health *n* = 331 (%)	Physicians *n* = 300 (%)	*p*-value
Gender					
Female	68.8%	1487 (74.8%)	193 (58.3%)	123 (41.0%)	< 0.001
Male	31.2%	502 (25.2%)	138 (41.7%)	177 (59.0%)	
Years of Experience					
< 5 years	16.4%	368 (18.5%)	47 (14.2%)	16 (5.3%)	< 0.001
5–10 years	37.7%	798 (40.1%)	128 (38.7%)	61 (20.3%)	
> 10 years	45.9%	823 (41.4%)	156 (47.1%)	223 (74.3%)	
Practicing in Abu Dhabi					
< 5 years	53.4%	1123 (56.5%)	154 (46.5%)	123 (41.0%)	< 0.001
5–10 years	26.8%	507 (25.5%)	108 (32.6%)	86 (28.7%)	
> 10 years	19.8%	359 (18.0%)	69 (20.9%)	91 (30.3%)	
Practice Setting					
Hospitals	76.4%	1507 (75.8%)	257 (77.6%)	238 (79.3%)	< 0.001
Clinics	10.3%	232 (11.7%)	15 (4.5%)	24 (8.0%)	
Medical and Surgical Centers	5.0%	61 (3.1%)	34 (10.3%)	35 (11.7%)	
Homecare/Long-term/Rehabilitation	8.3%	189 (9.5%)	25 (7.6%)	3 (1.0%)	
Type of CME/CPD Attended					
Live Online	48.5%	966 (48.6%)	171 (51.7%)	134 (44.7%)	< 0.001
Live In-person	43.6%	855 (43.0%)	136 (41.1%)	152 (50.7%)	
Recorded/Enduring	7.9%	168 (8.4%)	24 (7.2%)	14 (4.7%)	
Location of CME/CPD Attended					
Abu Dhabi	83.5%	1748 (87.9%)	239 (72.2%)	201 (67.0%)	< 0.001
Other Emirates	8.7%	83 (4.2%)	72 (21.8%)	72 (24.0%)	
Outside UAE	7.8%	158 (7.9%)	20 (6.0%)	27 (9.0%)	
Biggest Obstacles in CME/CPD					
Taking time off	38.4%	770 (38.7%)	82 (24.8%)	155 (51.7%)	< 0.001
Searching for CME/CPD	29.3%	588 (28.1%)	126 (38.1%)	53 (17.7%)	
Expense	20.1%	373 (18.8%)	91 (27.5%)	62 (20.7%)	
Travel time	6.4%	132 (6.6%)	19 (5.7%)	16 (5.3%)	
Poor CME Quality	5.8%	126 (6.3%)	13 (3.9%)	14 (4.7%)	

Hospital settings include general and specialized hospitals. Medical Centers refer to multispecialty centers with both medical and surgical services. Clinic settings include ambulatory outpatient facilities. Homecare/Long-term includes rehabilitation services, home healthcare, and long-term care facilities

*CME *Continuing Medical Education, *CPD *Continuing Professional Development, *UAE *United Arab Emirates

Values are presented as *n* (%). *P*-values were calculated using chi-square tests of independence

The analysis of perceived CPD learning needs revealed a distinct set of priorities among the surveyed healthcare professionals (Table 2). The top five CME topics identified by the overall cohort were the management of communicable diseases (39.1%), pain management (27.7%), procedural skills (25.5%), assessing psychosocial needs of patients (25.0%), and children’s health and wellbeing (24.2%). The management of communicable diseases consistently emerged as the primary topic of interest across all healthcare professional categories, accounting for 44.4%, 37.8%, and 35.0% for nurses, allied health professionals, and physicians, respectively. Notably, both communicable diseases and pain management as a CME need remained consistent across the three health professional subgroups, with only variations in the ranking order within the top five. Physicians and nurses identified procedural skills and the management of child health and wellbeing as top priorities. Meanwhile, nurses and allied health professionals emphasized the importance of assessing patients’ psychosocial needs, which represented 30.8% and 26.9% of their learning needs, respectively.

**Table 2:** Perceived CME and CPD learning needs among healthcare professionals in Abu Dhabi

Variables	Total Group Rank (Average %)	Nurses (*n* = 1989) Rank (%)	Allied Health (*n* = 331) Rank (%)	Physicians (*n* = 300) Rank (%)
Top 5 perceived CME needs (Group and Subgroup)				
Communicable and infectious diseases	**1st** **(39.1%)**	**1st** **(44.4%)**	**1st** **(37.8%)**	**1st** **(35.0%)**
Pain management	**2nd (27.7%)**	**3rd (33.6%)**	**4th (19.9%)**	**2nd** **(29.7%)**
Procedural skills	**3rd (25.5%)**	**2nd** **(36.3)**	11th (12.1)	**3rd** **(28.0%)**
Assessing patients’ social/psychological needs	**4th (25.0%)**	**4th (30.8%)**	**3rd (26.9%)**	6th (17.3%)
Childhood health and wellbeing	**5th (24.2%)**	**5th (30.4%)**	6th (17.8%)	**5th** **(24.3%)**
Patient safety and medical error	**-**	6th (28.8%)	**2nd** **(29%)**	14th (13.3%)
Management of common chronic conditions	**-**	7th (26.2%)	7th (17.5%)	**4th** **(24.7%)**
Mental Health	**-**	10th (17.9%)	**5th** **(19%)**	8th (14.7)
Top 5 perceived CPD needs (Group and Subgroup)				
Healthcare system	**1st (38.2%)**	**2nd (37.0%)**	**1st (42.9%)**	**2nd (34.7%)**
Communication skills	**2nd (34.3%)**	**1st (42.1%)**	**2nd (33.5%)**	**4th (27.3%)**
Applying evidence-based guidelines	**3rd (33.5%)**	**3rd (28.2%)**	**3rd (26.0%)**	**1st (46.3%)**
Stress management (self and others)	**4th (23.2%)**	**4th (25.3%)**	**4th (25.7%)**	6th (18.7%)
Leadership skills	**5th (20.9%)**	**5th (24.2%)**	**5th (22.4%)**	8th (16.0%)
Medical research	-	14th (10.4%)	11th (13.9%)	**3rd (33.0%)**
Academic Medicine	-	26th (6.7%)	23rd (7.3%)	**5th (21.0%)**

Values represent the percentage of respondents in each group who identified the topic as a priority learning need. For the whole cohort, topics are ranked based on overall prevalence across all professionals, calculated as the average percentage (%)

For CME needs, respondents selected from 33 clinical and specialty topics. For CPD needs, respondents selected from 34 professional development areas. The top five priorities for each professional group are shown in bold

*CME* Continuing Medical Education; *CPD *Continuing Professional Development

The analysis of perceived CPD learning needs, on the other hand, unveiled a unique set of priorities among the surveyed healthcare professionals. The overall cohort identified the following top five CPD topics: healthcare system (38.2%), communication skills (34.3%), applying evidence-based guidelines to practice (33.5%), stress management (23.2%), and leadership skills (20.9%) (Table 2). The top five learning needs were remarkably consistent across nurses and allied health professionals, except for two specific areas among physicians’ pattern of preferences. For physicians, applying evidence-based guidelines to practice emerged as the top priority (46.3%), followed by the healthcare system (34.4%). Communication skills ranked fourth (27.3%) among physicians, contrasting with its second-place position in the overall ranking. Interestingly, two CPD topics unique to the physician subgroup surfaced: medical research, ranking third (33.0%), and academic medicine, ranking fifth (21.0%).

Among the participants surveyed, the most prominent barrier to engaging in CME and CPD was the difficulty of taking time off from work, which was cited by 38.4% of respondents as their primary challenge. This was closely followed by the struggle to identify appropriate and relevant learning activities, which accounted for 29.3% of the reported limitations. Financial constraints also played a significant role, with 20.1% of participants highlighting expenses as a major hindrance. Travel time was less frequently mentioned, representing only 6.4% of the barriers, while the perceived quality of available learning activities was the least cited concern, contributing to just 5.8% of the limitations. The data also revealed significant differences among professional groups. Nurses and physicians predominantly identified taking time off as their most significant obstacle, whereas allied health professionals faced greater challenges in locating suitable CME/CPD opportunities that aligned with their needs.

## Discussion

This study represents the first comprehensive assessment of perceived CME/CPD needs among healthcare professionals in this region, providing crucial insights into the educational priorities of a diverse healthcare workforce. The significance of this research is particularly noteworthy given the unique characteristics of Abu Dhabi’s healthcare system, which, like other Gulf countries, relies on an expatriate workforce that includes professionals from more than 100 countries [11]. This cultural and educational diversity presents both challenges and opportunities for CME/CPD programming, making this research especially valuable for understanding and addressing the learning needs of such a diverse healthcare community.

**Fig. 1:**
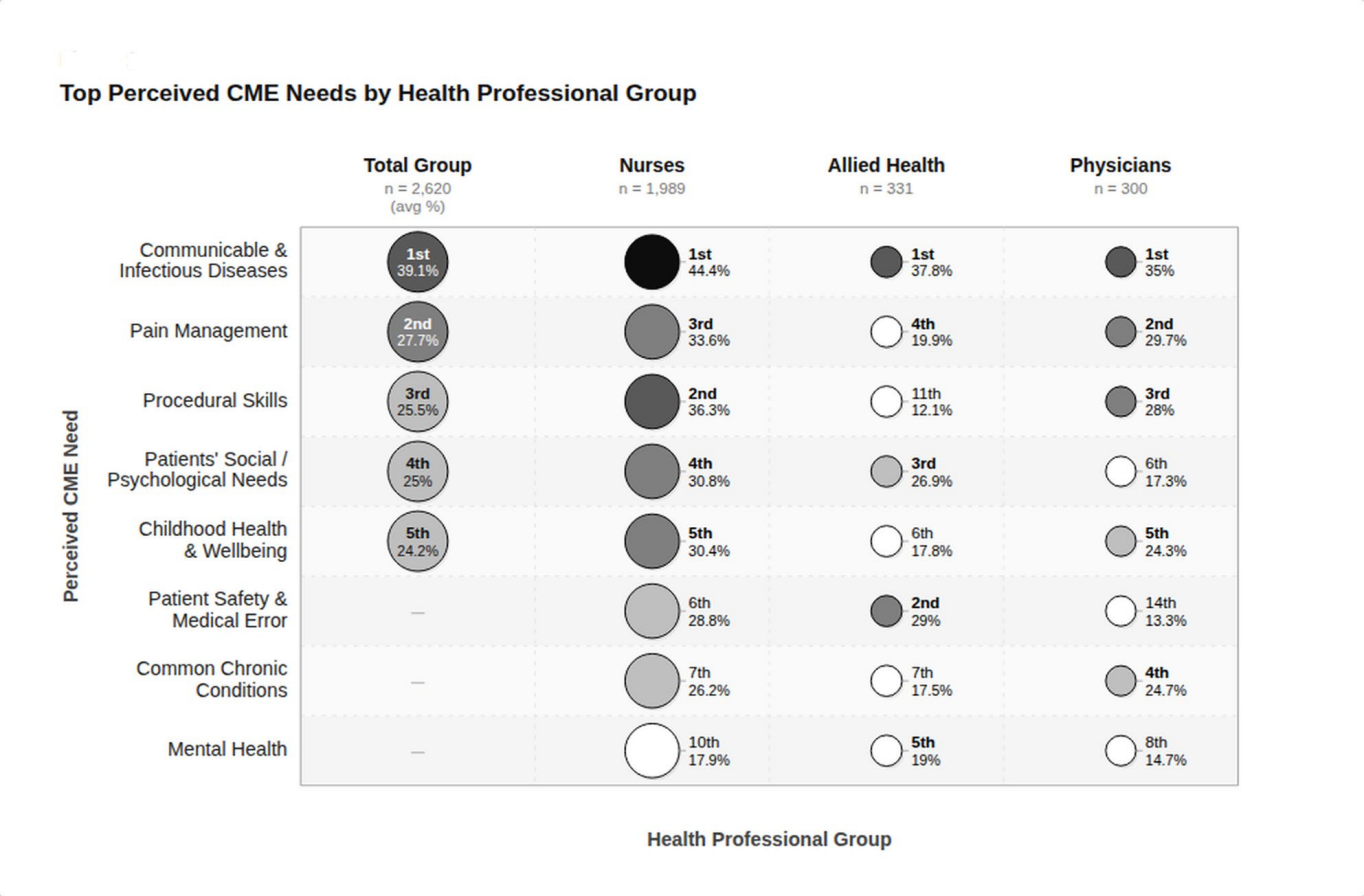
Perceived continuing medical education (CME) needs among three healthcare professional groups in Abu Dhabi; Nurses (n = 1,989), Allied Health professionals (n = 331), and Physicians (n = 300), alongside the total group average (n = 2,620). Each bubble represents a CME topic for a given professional group. Bubble size is proportional to the subgroup sample size. Bubble shading reflects the percentage of respondents within that group who identified the topic as a priority CME need, ranging from white (<20%) through light grey, mid grey, dark grey, to black (>41%). The number displayed within or beside each bubble indicates the topic's rank within that professional group. Bold-shaded bubbles denote topics ranked within the group's top 5 perceived CME needs; lighter bubbles indicate topics ranked outside the top 5. A dash (–) indicates the topic did not feature in the Total Group's top-5 average ranking. CME: Continuing Medical Education

Our findings revealed both consistency and distinct CME needs across professional categories, though with varying priorities within the top five topics (Fig. 1). Management of communicable diseases emerged as the primary concern across all professional categories, likely reflecting the ongoing global health challenges and the need for up-to-date knowledge in this rapidly evolving field [12]. Recent studies corroborate this finding highlighting the importance of infectious disease management in the wake of global health pandemics [13]. Pain management emerged as a high-priority CME need, especially for nurses and physicians, reflecting their direct patient care roles and aligning with previous research [6]. Studies emphasize the need for comprehensive, interprofessional pain education, addressing both pharmacological and biopsychosocial aspects of pain management to improve patient care outcomes [14].

The third-ranked topic, procedural skills (25.5%), represents a fundamental cornerstone of healthcare professional identity and an ethical obligation linked to healthcare outcomes. This topic ranked in the top 5 for nurses and physicians but 11th for allied health professionals, reflecting their different interventional roles [15]. Similarly, psychosocial assessment of patients (25%) ranked higher among allied health professionals (3rd) and nurses (4th) than physicians (6th), aligning with healthcare’s evolution toward patient-centered models addressing both physical and psychological dimensions. Research shows that thorough psychosocial assessment improves patient outcomes and satisfaction, confirming its essential role in high‑quality care [16]. Childhood health and wellbeing (24.2%) emerged as the fifth highest priority in the overall analysis, with consistent importance across professional groups. This topic received equal emphasis from nurses (30.4%) and physicians (24.3%), while allied health professionals ranked it sixth (17.8%) in their priorities. Uniquely, this was the only patient population-related topic that received high prioritization in learning needs across all health professional groups, standing out compared to women’s, men’s, and elderly health. Without detailed sub-analysis data regarding the specific scopes of practice among these health professionals, the underlying reasons for this consistent high prioritization across all three groups remain unclear.

**Fig. 2:**
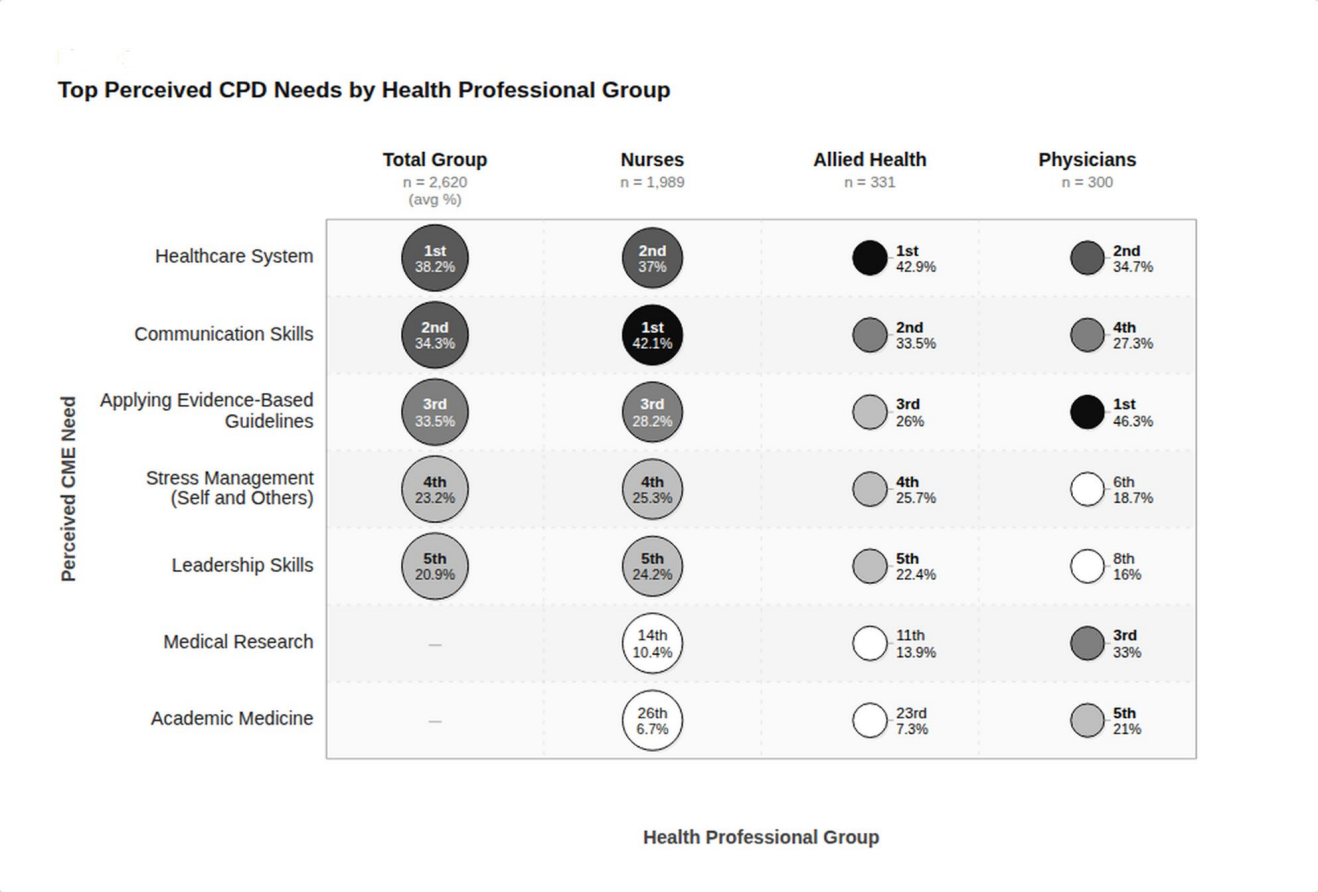
Perceived continuing professional development (CPD) needs among three healthcare professional groups in Abu Dhabi ; Nurses (n = 1,989), Allied Health professionals (n = 331), and Physicians (n = 300), alongside the total group average (n = 2,620). Each bubble represents a CPD topic for a given professional group. Bubble size is proportional to the subgroup sample size. Bubble shading reflects the percentage of respondents within that group who identified the topic as a priority CPD need, ranging from white (<20%) through light grey, mid grey, dark grey, to black (>41%). The number displayed within or beside each bubble indicates the topic's rank within that professional group. Bold-shaded bubbles denote topics ranked within the group's top 5 perceived CPD needs; lighter bubbles indicate topics ranked outside the top 5. A dash (–) indicates the topic did not feature in the Total Group's top-5 average ranking. CPD: Continuing Professional Development

Our assessment of CPD needs highlighted a strong interest in systemic and interpersonal competencies, which are essential for delivering effective patient care. With the exception of two specific areas among physicians, the top five learning needs were remarkably consistent across nurses and allied health professionals (Fig. 2).

Knowledge of the healthcare system emerged as the highest priority overall, cited by 38.2% of respondents. The high prioritization of healthcare system knowledge may be particularly relevant given that 53.4% of respondents had less than five years of experience in the Abu Dhabi health system. This finding is consistent with recent studies that underscore the growing importance of systems thinking in medical education [17, 18].

Communication skills were identified as the second most critical CPD need, with 34.3% of respondents emphasizing their importance. This finding underscores the increasing acknowledgment of effective communication as a fundamental element of healthcare professional competency. The prioritization of communication skills reflects the complexities of providing care in multicultural settings, where clear and empathetic communication is vital for ensuring patient safety and satisfaction. In the UAE and the broader region, communication has been established as a key pillar within professionalism frameworks, as evidence suggests that enhanced communication skills contribute to improved patient outcomes, a reduction in medical errors, and higher levels of patient satisfaction [19, 20].

The application of evidence-based practice (EBP) was ranked third overall in CPD needs, with 33.5% of participants identifying it as a priority. Physicians, in particular, demonstrated a significantly higher level of interest, with 46.3% emphasizing its importance. This strong focus on evidence-based practice highlights a broader shift toward data-driven decision-making in clinical settings and underscores the necessity for healthcare professionals to remain updated with the rapidly advancing field of medical knowledge. When combined with the top-priority learning need of “health system” knowledge, this reflects a comprehensive approach to clinical care in the modern era. In this context, healthcare professionals are expected to not only excel in clinical decision-making but also understand how these decisions fit within the broader health system. This dual competency ensures that patient care is both evidence-based and systemically informed, enabling healthcare providers to deliver more effective, efficient, and contextually appropriate care in an increasingly complex healthcare environment [21] .

Stress management (23.2%) and leadership skills (20.8%) completed the top five CPD needs, reflecting the challenging nature of healthcare professions and the need for strong leadership capabilities. The emphasis on stress management reflects the high-pressure healthcare environment and burnout’s impact on care quality [6].

While nurses and allied health professionals share similar priorities in their perceived CPD needs, physicians stand out with a distinct focus on competencies in medical research and academic medicine, both of which rank among their top five CPD priorities. This emphasis aligns with the significant growth of medical education in the UAE over the past decade, particularly in Abu Dhabi, where academic centers have become increasingly integrated into the healthcare system [11, 22]. Additionally, research has taken center stage in Abu Dhabi’s health sector, driven by substantial investments from both government and private entities in advanced biomedical research, health informatics, and digital innovation. These developments reflect a broader commitment to fostering a culture of research and academic excellence, which is now reflected in the CPD needs of physicians as they seek to stay at the forefront of biomedical research and innovation [23].

The study identified key limitations and barriers to conducting CME/CPD activities among healthcare professionals in Abu Dhabi. Time constraints emerged as the primary barrier, affecting 38.4% of participants particularly physicians and nurses a finding consistent with research across different training environments and regions [24–27].

Despite growing CME availability in the emirate, locating appropriate educational opportunities was the second most frequently reported challenge. Financial costs and travel requirements were identified as the third and fourth most common obstacles to participation in Abu Dhabi’s CME activities. Notably, quality concerns were minimal, with only 5.8% of respondents citing this as an issue, demonstrating substantial improvements in educational delivery resulting from both provider efforts and effective regulatory oversight [6].

The survey data indicated that a substantial majority of participants (92.1%) expressed a preference for live training formats, with nearly half (48.5%) favoring virtual platforms and 43.6% opting for in-person sessions. This trend mirrors findings from a recent UAE-based study examining CME preferences among pharmacists [27]. The strong inclination toward live, interactive formats, particularly virtual ones, highlights healthcare professionals’ prioritization of real-time engagement, even as distance learning gains traction. In contrast, recorded or self-paced e-learning materials were preferred by only 7.9% of respondents. The limited adoption of enduring educational resources may reflect insufficient familiarity with such formats or the limited availability of these resources from regional educational providers. Further research is necessary to elucidate the factors contributing to the low uptake of these materials in this context.

This study presents several limitations that warrant consideration. The demographic composition and professional backgrounds of the cohort exhibited significant imbalances, potentially impacting the overall results. Nurses constituted a substantial majority, accounting for 75.9% of respondents, while hospital-based practitioners represented 76.4% of the sample. Furthermore, considerable variations were observed in the participants’ practical experience levels. These disproportionate representations may have skewed the findings and might not accurately reflect the comprehensive needs of healthcare professionals across diverse settings. However, it is important to note that the sample broadly mirrors the composition and diversity of academic and clinical backgrounds within the Abu Dhabi healthcare workforce. The cross-sectional design of this study presents a limitation, as it captures only a specific moment in time. To address this, future research should consider longitudinal studies that monitor the evolution of healthcare professionals’ needs over time. Despite these limitations, the study provides valuable insights into the current state of healthcare professional needs in Abu Dhabi, serving as a foundation for future research and potential improvements in continuing education and professional development programs.

## Conclusion

This study provides comprehensive insights into the CME/CPD needs of healthcare professionals within Abu Dhabi’s diverse healthcare system. The findings reveal both shared and profession-specific learning priorities, highlighting the importance of tailored educational approaches while identifying common ground for interprofessional education initiatives. The clear emergence of communicable disease management, patient safety, and healthcare system knowledge as top priorities reflects modern healthcare challenges and opportunities. The identified barriers to participation and preferred learning modalities provide valuable guidance for future educational program development. These insights will be instrumental in shaping effective professional development strategies that support the delivery of high-quality healthcare in culturally diverse health systems, not only in Abu Dhabi but potentially in similar healthcare contexts globally. To strengthen future research, we recommend stratified sampling to ensure balanced representation across professional groups and practice settings, and to further evaluate the impact of tailored educational programs and innovative strategies to overcome participation barriers. 

## Supplementary Information

Supplementary Material 1.

## Data Availability

The datasets used and/or analyzed during the current study are available from the corresponding author on reasonable request.
