# Supplementary material for: Bridging knowledge gaps: identifying key educational priorities for healthcare professionals in the United Arab Emirates
Source: BMC Med Educ. 2026 Apr 23;26:925. doi: 10.1186/s12909-026-09121-x (PMC13238115; doi:10.1186/s12909-026-09121-x)
Supplement: Supplementary file 1 — Supplementary Material 1. [file 12909_2026_9121_MOESM1_ESM.pdf]

Ref : DOH/MRDD/2023/1142
Date : 06/07/2023

Dr. Hatem Faraj Al Ameri
Manager Medical Education & Examination, Healthcare Licensing & Medical Education
Department of Health - Abu Dhabi

Subject: Medical Research and Development Division – Exemption Letter

Study Title:
The Continuous Medical Education Needs assessment survey for Health Professional

Thank you for submitting your application to Medical Research and Development Division. Your proposal was evaluated in light of the DOH regulations that govern the protection of human research subjects.

Please note that your proposal is exempted from Abu Dhabi Health Research and Technology Committee (ADHRTC) review, your proposed project employs surveys that pose no more than minimal risk to the participants. The information will be obtained in such a way that one's responses will not be linked to one's identity or identifying information. Moreover, accidental disclosure of the participants' responses would not have the potential to harm to the person's reputation, employability, financial status, or legal standing. For these reasons, the Medical Research and Development Division has determined that your proposed study is exempt from Abu Dhabi Health Research and Technology Committee (ADHRTC) review process.

Even though your project is exempt from Abu Dhabi Health Research and Technology Committee (ADHRTC) review process, the research must be conducted according to the DOH standards and guidelines and not provide:

- information obtained is recorded in such a manner that human subjects can be identified, directly or through identifiers linked to the subjects; and
- any disclosure of the human subjects' responses outside the research could reasonably place the subjects at risk of criminal or civil liability or be damaging to the subjects' financial standing, employability, or reputation.

Yours Sincerely,

Rasheed Alhammadi
Division Manager Medical Research & Development

Cc:

PO Box 5674 Abu Dhabi, U.A.E
+971 2 4493333 +971 2 4449822 doh.gov.ae
